# Bio-Assay-Guided Study of *Chaenomeles japonica*–Cytokine Modulation by Fruit Aqueous Extract In Vitro in Connection with Its Processing with Enzymatic and Microbial Additives

**DOI:** 10.3390/nu17233716

**Published:** 2025-11-27

**Authors:** Agata J. Olędzka, Aleksandra Sirak, Dariia Hovtvian, Oleh Koshovyi, Monika E. Czerwińska

**Affiliations:** 1 Department of Biochemistry and Pharmacogenomics, Faculty of Pharmacy, Medical University of Warsaw, 1 Banacha Str., 02-097 Warsaw, Poland; agata.oledzka@wum.edu.pl; 2 Centre for Preclinical Research, Medical University of Warsaw, 1B Banacha Str., 02-097 Warsaw, Poland; 3 Doctoral School, Medical University of Warsaw, 61 Zwirki i Wigury Street, 02-091 Warsaw, Poland; 4 Student Scientific Association, Department of Biochemistry and Pharmacogenomics, Faculty of Pharmacy, Medical University of Warsaw, 1 Banacha Str., 02-097 Warsaw, Poland; 5 Department of Pharmacognosy, National University of Pharmacy, 53 Pushkinska Str., 61002 Kharkiv, Ukraine; 6 Institute of Pharmacy, Faculty of Medicine, University of Tartu, Nooruse 1, 50411 Tartu, Estonia

**Keywords:** cytokines, inflammation, post-processing metabolites, microbiota processing, Rosaceae

## Abstract

**Background/Objectives**: Phytochemicals from *Chaenomeles japonica* (CJ) (Thunb.) Lindl. ex Spach, a plant belonging to the Rosaceae family, are recognized for their potential to inhibit enzymes associated with diabetes, obesity, neurodegeneration, and inflammation. However, the influence of constituents from different plant parts on cytokine secretion has not yet been explored or comparatively analyzed. **Methods**: This study aimed to evaluate the anti-inflammatory potential of CJ by assessing its effects on chemokine and cytokine secretion, including interleukin (IL)-8, IL-1β, TNF-α, IL-6, and IL-10. Extracts from various plant parts (fruit, seed, flower, and leaf) were examined for their ability to modulate cytokine production in human neutrophils (PMNs). Among them, the aqueous fruit extract exhibited the strongest activity and was subsequently tested on peripheral blood mononuclear cells (PBMCs) and the human intestinal epithelial cell line Caco-2. The extract was also subjected to in vitro gastrointestinal digestion to assess the stability and bioactivity of its metabolites. The phytochemical composition of CJ preparations was characterized by ultra-high-performance liquid chromatography coupled with diode-array detection and tandem mass spectrometry (UHPLC-DAD-MS/MS). **Results**: The aqueous fruit extract significantly reduced the secretion of pro-inflammatory cytokines across all tested models. Fractions obtained after in vitro digestion also inhibited IL-8 release in Caco-2 cells. **Conclusions**: The most active fractions were rich in flavan-3-ols and proanthocyanidins. These findings indicate that CJ fruit possesses notable anti-cytokine properties and may serve as a promising natural source for developing functional food.

## 1. Introduction

*Chaenomeles japonica* (Thunb.) Lindl. ex Spach. (CJ), commonly known as Japanese quince and belonging to Rosaceae, has been used in Traditional Chinese Medicine (TCM) for thousands of years to treat various diseases, from the common cold and sore throat to mastitis. After its introduction to Europe in the nineteenth century, the plant was used at first as an ornamental plant. As recently revised by Marat et al. (2022), Japanese quince shrub’s cultivation and breeding in Lithuania, Latvia, and Sweden were developed within the cultivation program in the 1990s [1]. In that time, the fruit shrub cropland in these regions reached 400 ha and spread to Estonia, Finland, and Poland [1]. As the CJ fruits are sour and bitter in taste, they are consumed after processing, in the form of juices, jams, wines, and liquors, which are often homemade [1,2].

Inflammation is an innate immune response triggered by harmful stimuli, playing a key role in developing numerous diseases when continuously overactivated [3]. Chronic inflammation contributes to diabetes, asthma, cancer, heart disease, atherosclerosis, and autoimmune disorders. Under normal conditions, the intestinal epithelium, covered by mucus, serves as a critical barrier against gut microbiota [3]. Pathogen-associated molecular patterns (PAMPs), including lipopolysaccharides (LPSs) from Gram-negative bacteria, can pass through when this barrier is compromised, leading to immune responses. Neutrophils (polymorphonuclear leukocytes, PMNs) and monocytes/macrophages, a network of the first line of defense in the gut barrier, produce inflammatory mediators to combat invading pathogens. In inflammatory bowel diseases (IBDs) like Crohn’s disease and ulcerative colitis, an abundance of neutrophils in intestinal lesions is observed, further highlighting the immune response in inflammation [4]. During the 20th century, IBDs were almost exclusively recognized in industrialized areas of Western and Northern Europe, North America, Australia, and New Zealand. However, over the past 50 years, IBDs have spread into developing countries in Asia, Latin America, South America, and Africa. In addition, the incidence of childhood IBD continues to rise. Approximately 25% of patients with IBD will suffer from it before 20 years of age [5].

Neutrophils and macrophages secrete cytokines and chemokines, acute-phase proteins, and vasoactive amines in response to stimuli. The overactivation of neutrophils and their trafficking to other tissues in a chemoattractant-driven pathway is thought to contribute to the development of intestinal inflammation [6,7,8]. There is increasing evidence of tissue cross-talk via inflammatory mediators, signaling lipids, and extracellular vesicles [9]. Therefore, developing more complex models is increasingly warranted to replicate bidirectional cellular interactions.

Plant-derived preparations utilize various materials like leaves, flowers, or fruits, and are consumed daily as part of a diet. Optimizing bio-resources for phytochemistry and pharmacological activity is essential, with bio-assay-guided investigations aiding in identifying the most valuable sources of active compounds. Japanese quince contains phytochemicals such as flavonoids, proanthocyanidins, triterpenes, organic acids, and fibers, which may validate its medicinal and industrial uses [10,11]. However, the presence of these compounds varies between the seeds, leaves, and fruits [2,12,13,14,15]. So far, their extracts have demonstrated antioxidant activity, a chemoprotective effect on malignant cells, and a reduction in inflammatory mediators, including nuclear factor (NF)-κB in certain cell models [15,16,17,18]. In this study, we focused on screening various plant parts (leaf, flower, seed, and fruit), which have never been compared to establish which is the most valuable and provides the most relevant future perspectives. Apart from some data on fruit, for which research is in progress, it is worth indicating whether any plant materials derived and wasted from Japanese quince cultivation might be useful due to the activity of their constituents. In our study, the anti-inflammatory effects of different plant extracts and post-digestive fractions of fruit aqueous extract were tested in PMN, peripheral blood mononuclear cells (PBMCs), and a human adenocarcinoma cell line (Caco-2) to evaluate cytokine and chemokine release. Considering the limited bioavailability of phytochemicals in raw plant materials, our research highlighted the role of digestion in shaping plant metabolite activity, which is crucial to understanding their behavior in the human digestive system, bioaccessibility, and therapeutic as well as nutritional potential. It is also believed that the utility of advanced microfluidic conditions is a promising strategy for research in the complex field of microbiota–human cell communication [19]. Our study highlights the anti-inflammatory potential of CJ extracts, particularly fruit ones, emphasizing their ability to modulate inflammatory pathways and their promise for therapeutic applications as a functional food.

## 2. Materials and Methods

### 2.1. Reagents

Sodium citrate tribasic dihydrate (C7254), citric acid (CHEM-115382101), glucose (114595600) for citrate dextrose solution (ACD), and dimethyl sulfoxide (DMSO, 113635509) were purchased from Chempur (Piekary Śląskie, Poland). The water purification system Millipore Simfilter Simplicity UV (Molsheim, France) was used to obtain ultra-pure water. Dextran from *Leuconostoc mesenteroides* (31398-500 G), propidium iodide (PI, P4170-10 MG), and dexamethasone (Dex, D1756-25 MG), amphotericin B (A2942-100 ML), Roswell Park Memorial Institute (RPMI) 1640 medium (R7509-500 ML), and 3-(4,5-dimethylthiazol-2-yl)-2,5-diphenyltetrazolium (MTT, 0793-5 G) were purchased from Sigma-Aldrich (Saint Louis, MO, USA). Penicillin–streptomycin (L0022-100) and Dulbecco’s Modified Eagle Medium (DMEM, L0103-500) enriched with 3.7 g/L NaHCO3 and 4.5 g/L glucose, with stable L-glutamine, low endotoxin, fetal bovine serum (FBS, S1860-500), and phosphate-buffered saline (PBS, L0615-500) were purchased from Biowest (Nauillé, France). Pancoll Human (1.077 g/mL, P04-601000) was from PAN-Biotech (Aidenbach, Germany). (Ca2+)-Free PBS (L0615) was purchased from BioWest (Nuaillé, France). Recombinant human IL-1β protein (IL038), LPS (2552117) from *Escherichia coli* (0111:B4), and formic acid (100264) were obtained from Merck (Darmstadt, Germany). TrypLE^TM^ Express Enzyme (12604021) and gentamicin (15710-049) were purchased from Gibco Thermo Fisher (Waltham, MA, USA). Recombinant TNF-α (300-01A), as well as recombinant interferon (IFN)-γ (300-02), were purchased from PeproTech (USA). Sets of immunosorbent-assay for human IL-8 (555244), TNF-α (555212), and IL-1β (557953), IL-6 (555220), and IL-10 (555157) were purchased from BD Biosciences (Erembodegem, Belgium). Salivary α-amylase (A0521-2.5KU, 170.1 units/mg), pepsin (P1725-100G, >400 U/mg protein), pancreatin from porcine pancreas (P1750-100G), and bile extract porcine (B8631-100G) from Sigma-Aldrich Chemie GmbH (Steinheim, Germany) were used. Ethyl acetate (141-78-6) was purchased from POCH (Gliwice, Poland). Brain heart infusion (BHI, P-0051) was purchased from BTL (Łódź, Poland). Buffy coats, used for PMN and PBMC isolation, were purchased from the Warsaw Blood Donation Centre (Warsaw, Poland). The Caco-2 cell line (ACC-169) originated from the DSMZ German Collection of Microorganisms and Cell Cultures, Leibnitz Institute (Braunschweig, Germany).

### 2.2. Plant Material Collection and Extract Preparation

Raw plant materials of *Chaenomeles japonica* (Thunb.) Lindl. ex Spach var. alpina were collected in October 2018 in the Botanical Garden of the Center for Biological Diversity Conservation in Powsin (Polish Academy of Sciences, Poland) (52°06′17′′ N, 21°05′42′′ E) (Figure S1).

Four parts of the *Chaenomeles japonica* were used (leaves, flowers, seeds, and fruits without seeds), and two types of extracts, ethanolic (60%, *v*/*v*) and aqueous (ratio 1:20 plant material to solvent, *m*/*v*), were used in the study. The extracts were dissolved in distilled water (Simplicity UV Millipore, Molsheim, France) and sonicated in an ultrasonic bath Polsonic (Warsaw, Poland). Afterward, the extracts were freeze-dried in Telstar Cryodos 50 (Terassa, Spain) and stored in the fridge. For phytochemical analysis, the stock solutions were prepared in a mixture of mobile phases (0.1% HCOOH in water and acetonitrile, 1:1, *v*/*v*) before use. In the case of biological assays, the extracts were dissolved in a mixture of DMSO and deionized water (1:1, *v*/*v*) to achieve a concentration of 20 mg/mL (stock solution) and stored in the freezer. Before each experiment, the appropriate concentrations were prepared in the medium. The concentration of DMSO did not exceed 0.5% in the well.

### 2.3. Phytochemical Analysis 

#### 2.3.1. UHPLC-DAD-MS/MS Conditions

UHPLC-DAD-MS^n^ analysis was performed on a UHPLC-3000 RS system (Dionex, Sunnyvale, California, CA, USA) with DAD detection and an AmaZon SL ion trap mass spectrometer with ESI interface (Bruker Daltonik GmbH, Bremen, Germany). Separation was performed on a Zorbax SB-C18 column (150 × 2.1 mm, 1.9 μm; Agilent, Santa Clara, CA, USA), set at 25 °C. For preliminary phytochemical analysis of extracts, the mobile phase (A) was 0.1% HCOOH in water, and the mobile phase (B) was 0.1% HCOOH in acetonitrile. A linear gradient system was used: 0–60 min. 4–26% B; 60–90 min. 26–95% B. The flow rate was 0.2 mL/min. The column was equilibrated for 10 min between injections. UV spectra were recorded over a range of 200 to 450 nm, and chromatograms were acquired at 240, 280, 325, and 350 nm. The LC eluate was introduced directly into the ESI interface without splitting. The nebulizer pressure was 40 psi; the dry gas flow was 9 L/min; the dry temperature was 300 °C; and the capillary voltage was 4.5 kV. Analysis was carried out using scans from *m*/*z* 200 to 2200. Compounds were analyzed in negative ion mode. The MS/MS was switched on and tuned to detect neutral loss of 162, 132, 152, 146, and 176 corresponding to the cleavage of sugars or phenolic acid moieties. The MS^2^ fragmentation was obtained for the most abundant ion at the time. The phytochemical analysis of CJ extracts was performed as it was previously described [20].

#### 2.3.2. Total Phenol Content

The sum of phenols (TPC) was determined using a spectrophotometric method with Folin–Ciocalteu’s reagent according to the assay previously described (Mainka et al., 2021) [21]. The absorbance was measured at 765 nm in the microplate reader (BioTek Epoch, Santa Clara, CA, USA). The results are expressed as gallic acid equivalent (μg GAE/mg d.w. of the extract).

#### 2.3.3. Total Flavonoid Content

The sum of flavonoids (TFC) was determined using a spectrophotometric method with 1% AlCl_3_ according to the assay previously described (Zulkifli et al., 2020) [22]. The absorbance was measured at 415 nm in the microplate reader (BioTek Epoch, Santa Clara, CA, USA). The results are expressed as quercetin equivalent (μg Q/mg d.w. of the extract).

### 2.4. Cytokine Secretion in PMN, PBMC, and Caco-2

The study was performed following the Declaration of Helsinki and the guidelines of the Ethical Committee of the Medical University of Warsaw regarding the use of human-derived material. The use of buffy coats does not require ethical approval, as the material is commercially available to purchase. The buffy coats were obtained from male, healthy donors before the age of 35. For each replicate of the experiments, fresh material was used. Faecal samples were donated by healthy volunteers (researchers) aged 30–38 with no medical history of gastrointestinal disorders. Researcher self-experimentation does not constitute human clinical trials research as defined by the Department of Health and Human Services or U.S. Food and Drug Administration regulations [23]. Considering the non-invasive nature of the study and the minimal safety risks involved, the research does not qualify as a medical experiment under local regulations. Therefore, in accordance with the policy of the Ethics Committee of the Medical University of Warsaw, ethical approval was not required (AKBE/210/2024). Donors had not used any antibiotics for the previous six months. Intake of flavonoid, tannin, and anthocyanin-containing products was forbidden for 4 days before sample collection.

#### 2.4.1. PMN Culture

Neutrophils were isolated by dextran sedimentation and centrifugation in a density gradient with buffer for leukocyte isolation (Pancoll Human) according to Böyum’s method [24]. The isolation was performed as previously described [25]. The cells were seeded in 96-well plates for cytokine/chemokine secretion assays (2 × 10^6^ cells/well). Both neutrophil cytotoxicity by propidium iodide staining and cytokine/chemokine secretion with enzyme-linked immunosorbent assays (ELISA) were evaluated according to previously described methods [26].

The cells were treated with aqueous and ethanolic extracts from CJ leaves, flowers, seeds, and fruits at concentrations of 5, 50, and 100 μg/mL (50 μL), before stimulation with 10 μL LPS (100 ng/mL) and incubated for a further 20 h in standard conditions (5% CO_2_, 37 °C). As positive controls, Triton X-100 at 0.1% (*v*/*v*) and DEX at concentrations of 0.01, 0.1, and 1 µM were used for cytotoxicity and cytokine/chemokine secretion evaluation, respectively. As a negative control, the cells were stimulated with LPS, but not treated with extracts. The PMN cytotoxicity was evaluated by flow cytometry with BD FACSVerse (BD Biosciences, San Jose, CA, USA) with PI (0.5 μg/mL) staining. The experiments were repeated at least three times in triplicate in all cellular models.

#### 2.4.2. PBMC Culture

The PBMCs were isolated according to the method originally described by Böyum [24]. A detailed process description has been previously provided [27]. The cells were seeded in 96-well plates for cytokine secretion assays (2 × 10^6^ cells/well). The PBMCs were treated with extracts at concentrations of 5, 50, and 100 μg/mL (50 μL) before stimulation with 10 μL LPS (100 ng/mL) and incubated for a further 48 h in standard conditions (5% CO_2_, 37 °C). Dexamethasone was used as a positive control at concentrations of 0.01, 0.1, and 1 µM. As a negative control, the cells were stimulated with LPS, but not treated with extracts. The PBMC cytotoxicity was evaluated by flow cytometry with BD FACSVerse (BD Biosciences, San Jose, CA, USA) with PI (0.5 μg/mL) staining as previously described [27]. Triton X-100 (0.1% *v*/*v*) was used as a positive control.

#### 2.4.3. Caco-2 Cell Culture

The human adenocarcinoma cells (Caco-2) were cultured in DMEM supplemented with 20% FBS, L-alanyl-L-glutamine (2 mM), penicillin (100 IU/mL), streptomycin (100 μg/mL), and amphotericin B (2.5 ng/mL). The detailed cell culture conditions were previously described [25]. In all in vitro experiments, passages between 20 and 45 were used. The Caco-2 cells were seeded in 24-well plates at a density of 2 × 10^5^ cells/well in DMEM with 20% FBS (*v*/*v*). The differentiation procedure (72 h) of adenocarcinoma cells into epithelial cells was started after 24 h incubation, by replacing the entire medium with DMEM supplemented with 5% FBS (*v*/*v*) and sodium butyrate (5 mM) [25].

The differentiated cells were incubated for 24 h in standard conditions in a humidified atmosphere with 5% CO_2_ at 37 °C, with aqueous CJ fruit extract at concentrations of 50, 100, and 500 μg/mL or fractions, obtained after its gastrointestinal (GI) digestion in vitro, at concentrations of 1, 5, 20, and 50 μg/mL. After that time, the solution composed of TNF-α (10 ng/mL), IL-1β (25 ng/mL), IFN-γ (10 ng/mL), and LPS (100 ng/mL) was added to the Caco-2 cells to stimulate the IL-8 secretion.

#### 2.4.4. The Enzyme-Linked Immunosorbent Assays

The ELISA assays were used to examine selected cytokine/chemokine secretion by PMN, PBMC, and Caco-2 cells. The ELISA assays for released IL-1β, IL-8, IL-6, IL-10, and TNF-α into cell supernatants were performed according to the manufacturer’s instructions. The effect on the cytokine or chemokine production was calculated as the percentage of released factor compared with LPS-stimulated control without the tested extract/fraction. As a positive control, Dex or prednisone was used.

### 2.5. Digestion Procedure In Vitro

**Figure 1:**
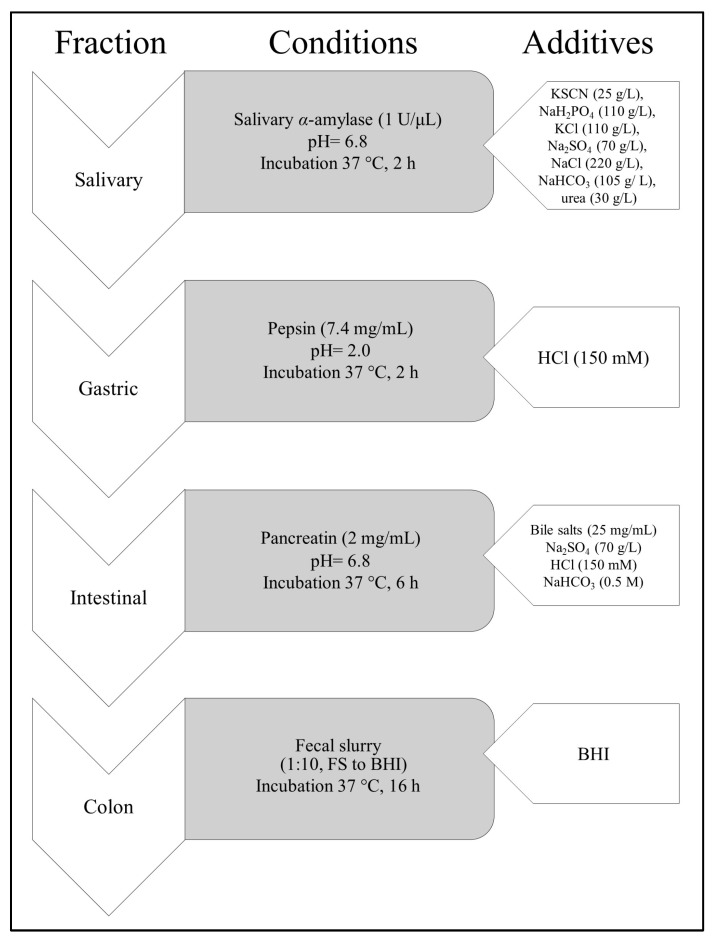
Scheme of gastrointestinal digestion in vitro of aqueous extract of CJ (*Chaenomeles japonica*) fruit.

The digestion procedure of aqueous CJ extract was performed according to the previously described modified methods [28,29]. The in vitro digestion procedure was performed as described [20]. To mimic the gastrointestinal tract conditions, digestion was performed in four compartments: salivary (S), gastric (G), intestinal (I), and colon (FS). The procedure was carried out with the presence (+E) or absence (−E) of gastrointestinal enzymes to assess whether digestive enzymes affect the changes of phytochemicals in the extracts. The general conditions and scheme of the study are presented in Figure 1. The CJ aqueous fruit extract was applied at 100 mg/mL starting concentration. The 5 mL aliquots from each compartment were extracted with ethyl acetate in a ratio of 1:10 (*v*/*v*), and next the solvent was evaporated under a vacuum. After the procedure, the samples were prepared for further testing of IL-8 secretion in Caco-2 cells.

### 2.6. Statistical Analysis

Each extract or fraction was tested in triplicate within three independent procedures. The results are expressed as means ± standard error of the mean (SEM). The experiments were performed using samples obtained from three independent donors. The values were normalized to the stimulated control. The statistical significance of the differences between means was established by testing homogeneity of variance and normality of distribution, followed by ANOVA and non-parametric methods such as the Kruskal–Wallis and Mann–Whitney U tests. The *p*-values below 0.05 were considered statistically significant. All analyses were performed using Statistica 13.3 (TIBCO Software Inc., Palo Alto, CA, USA).

## 3. Results

### 3.1. Phytochemical Analysis of CJ Extracts

**Figure 2:**
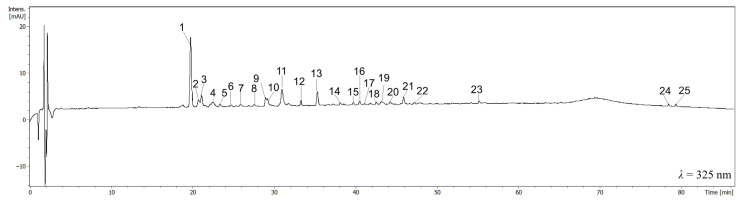
UV chromatogram of aqueous extract from fruits of CJ (10 mg/mL) registered at λ = 325 nm.

The phytochemical profile of leaves (Figure S2), flowers (Figure S3), seeds (Figure S4), and fruits (Figure 2 and Figure S5) showed qualitative differences in phenolic compounds. In general, significant qualitative differences were not observed between aqueous and ethanolic extracts. Although in the ethanolic extracts of leaves (Table S1) and flowers (Table S2), such compounds as caffeoylquinic acid derivative and epi (catechin) rhamnohexoside, respectively, were identified in contrary to the aqueous extracts. The most abundant compound of leaf and flower extracts was the dimer of caffeoylquinic acid (Tables S1 and S2). In addition, a few caffeoylquinic acid derivatives were detected, particularly in the leaf extracts. Apart from them, the glycosides of quercetin, kaempferol, and naringenin were tentatively assigned (Table S1). The major ion in the MS spectrum of kaempferol dirhamnoside is [M-H]^−^ *m*/*z* 577, which can also be found in the spectrum of procyanidin B-type dime [13]. However, the MS/MS spectrum of the precursor ion *m*/*z* 577 exhibited main fragmentary ions at *m*/*z* 431 and *m*/*z* 285, which should be rather assigned to kaempferol. Additionally, the UV spectrum (λ_max_ 264 and 343 nm) was more characteristic of flavonoids than proanthocyanidins. It is worth noting that we did not detect proanthocyanidins in the leaf extracts. Vomifoliol pentosylhexoside ([M-H]^−^ *m*/*z* 517) at R_t_ = 28.0 min was tentatively assigned in the leaf extracts based on the available literature [30]. Epicatechin ([M-H]^−^ *m*/*z* 289) at R_t_ = 28.1 min was tentatively assigned in the flower and fruit extracts. The ions from the MS^2^ fragmentation pattern of epicatechin were as previously described [31]. Moreover, gallic acid derivative was detected in the flower extracts (Table S2). In the case of its parent ion, after the loss of *m*/*z* 46 or *m*/*z* 152 and *m*/*z* 179, the fragmentary ions such as [M-H]^–^ *m*/*z* 294, [M-H]^–^ *m*/*z* 188, and [M-H]^–^ *m*/*z* 161 were detected. The loss of *m*/*z* 152 is characteristic of the galloyl compounds. Thus, a compound was described as a derivative of gallic acid. Proanthocyanidins were the fruit extracts’ most abundant class of compounds (Table 1). In the case of Fr_aq (Figure 2), apart from characteristic proanthocyanidins, some compounds were registered at R_t_ = 40.5 min ([M-H]^−^ *m*/*z* 720) and R_t_ = 41.8 min ([M-H]^−^ *m*/*z* 415). The fragmentary ions of precursor ion [M-H]^−^ *m*/*z* 720, such as *m*/*z* 575, *m*/*z* 489, *m*/*z* 451, and *m*/*z* 207 were registered in the negative ESI mode [32]. On the other hand, the ion [M-H]^–^ *m*/*z* 575 registered in the MS^2^ pattern in negative ESI mode may result from the loss of two protons by the procyanidin dimer. Last but not least, in the MS spectrum of extracts Sd_et and Sd_aq we found the main ion [M-H]^–^ *m*/*z* 456 at R_t_ = 32.2 min, or the ion [M-H]^–^ *m*/*z* 456 in the negative ESI mode was the most characteristic fragmentary ion of other compounds at R_t_ = 38.3 min [M-H]^–^ *m*/*z* 618 and R_t_ = 39.3 min [M-H+HCOOH]^–^ *m*/*z* 664 (Figure S4). In addition, the MS^2^ fragmentation pattern of [M-H]^–^ *m*/*z* 456 showed a signal at *m*/*z* 323 in the negative ionization mode. We tentatively assigned them amygdalin and hexosides of amygdalin rather than terpene derivatives (Table S3) [33].

### 3.2. The Quantitative Analysis of Extracts

The highest polyphenol content was determined in the leaf and flower extracts, and the lowest in the seed extracts (Table 2). It can be concluded that the great amount of polyphenols in the leaf extracts is flavonoid fraction, whereas no flavonoids are determined in the fruit extracts (Table 3). The results of the quantitative analysis confirm that the dominating polyphenols in fruit extracts are catechin derivatives, as established in HPLC-DAD-MS/MS analysis.

### 3.3. Cell Viability After Treatment with Chaenomeles japonica Extracts and GI Fractions

**Figure 3:**
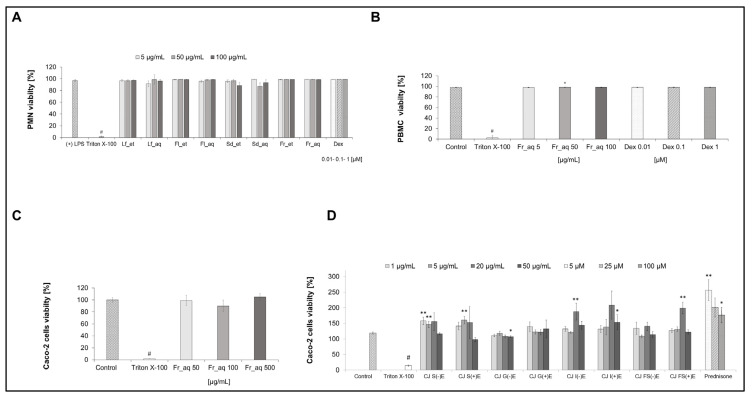
Viability of PMN treated with extracts from different parts of CJ (**A**), PBMC (**B**), and Caco-2 treated with CJ (*Chaenomeles japonica*) fruit extracts (**C**) or GI fractions of aqueous extract of CJ fruit (**D**). Lf_et—ethanolic extract of CJ leaves, Lf_aq—aqueous extract of CJ leaves, Fl_et—ethanolic extract of CJ flowers, Fl_aq—aqueous extract of CJ flowers, Sd_et—ethanolic extract of CJ seeds, Sd_aq—aqueous extract of CJ seeds, Fr_et—ethanolic extract of CJ fruits, Fr_aq—aqueous extract of CJ fruits; CJ fractions without digestive enzymes (−)E or with digestive enzymes (+)E: salivary (S), gastric (G), intestinal (I), colon (FS); Dex—dexamethasone; # *p* < 0.001 vs. control; * *p* < 0.05 vs. control, ** *p* < 0.001 vs. control.

The results of the viability of all cells used in this study after the treatment with extracts or GI fractions are shown in Figure 3. All aqueous and ethanolic CJ extracts from leaves, flowers, seeds, and fruits did not affect the PMN cell viability in the tested concentration range (Figure 3A). The aqueous CJ fruit extract did not affect the PBMC cell viability in the tested concentration range (Figure 3B). The aqueous CJ fruit extract (Figure 3C) and its GI fractions (Figure 3D) did not affect the Caco-2 cell viability in the tested concentration range.

### 3.4. Activity of Extracts from Different Parts of CJ in PMN After Chaenomeles japonica Extract Treatment

#### 3.4.1. TNF-α Secretion

**Figure 4:**
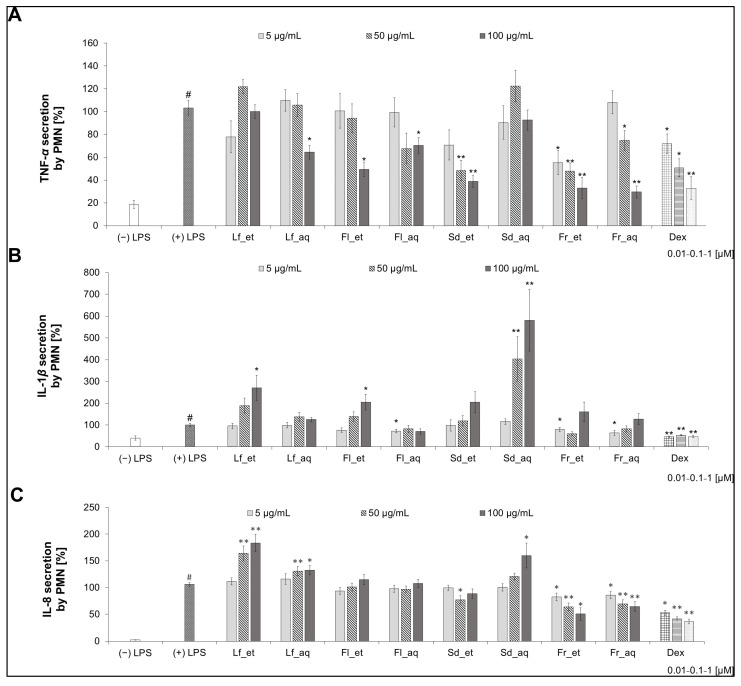
Effect of aqueous (aq) and ethanolic (et) extracts from leaves (Lf), flowers (Fl), seeds (Sd), and fruits (Fr) of CJ (*Chaenomeles japonica*) on the secretion of TNF-α (**A**), IL-1β (**B**), and IL-8 (**C**) in PMN. Lf_et—ethanolic extract of CJ leaves, Lf_aq—aqueous extract of CJ leaves, Fl_et—ethanolic extract of CJ flowers, Fl_aq—aqueous extract of CJ flowers, Sd_et—ethanolic extract of CJ seeds, Sd_aq—aqueous extract of CJ seeds, Fr_et—ethanolic extract of CJ fruits, Fr_aq—aqueous extract of CJ fruits; Dex—dexamethasone; # *p* < 0.001 vs. (−) LPS; * *p* < 0.05 vs. (+) LPS, ** *p* < 0.001 vs. (+) LPS.

The ethanolic CJ extracts from fruit and seed expressed the strongest ability to inhibit TNF-α secretion in a concentration-dependent manner, comparable to the effect of Dex (Figure 4A). The ethanolic extracts of seeds and fruits showed higher activity than aqueous ones. The most active extract in the whole concentration range was the Fr_et. The difference between the aqueous and ethanolic seed extract activity was the most relevant. The Sd_aq, Fl_et, Fl_aq, Lf_et, and Lf_aq extracts, with some exceptions at the highest concentrations, do not have any relevant activity in decreasing the TNF-α release in PMN.

#### 3.4.2. IL-1β Secretion

The CJ extracts in the highest concentration (100 μg/mL) tend to stimulate rather than inhibit the secretion of IL-1β in PMN (Figure 4B). The most significant effect can be observed for the Sd_aq—581.41 ± 142.15% (100 µg/mL). The most relevant inhibiting activity was observed in the aqueous extract from fruit at the concentration of 5 μg/mL, which showed the secretion of IL-1β at 64.12 ± 12.36% (*p* < 0.05), while the ethanolic extract from fruit at the concentration of 50 μg/mL caused the release of IL-1β at 59.79 ± 9.56% (*p* > 0.05). The effect of both fruit extracts was slightly weaker than Dex (from 45.68% to 53.28%). The Fl_aq affected the cytokine release similarly at each concentration. There are relevant differences between the ethanolic and aqueous extracts from the seed, flower, and leaf, whereas the effect of both fruit extracts is comparable. The aqueous extracts showed a tendency to stronger inhibition of IL-1β secretion.

#### 3.4.3. IL-8 Secretion

The aqueous extract from the CJ fruit had a relevant influence on decreasing the release of IL-8 in PMN (Figure 4C). A comparable effect could be observed for the ethanolic version of the extract (50.45 ± 11.89%), which was comparable with the effect of Dex (from 36.42% to 52.71%). Only those two extracts inhibited the chemokine secretion in a concentration-dependent manner, while Sd_aq, Fl_aq, Lf_et, and Lf_aq had an opposite effect, stimulating the secretion in a concentration-dependent manner. The ethanolic leaf and aqueous seed extract showed the strongest effect in increasing the IL-8 release.

Considering the relevant activity of CJ Fr_aq extract, it was selected for further investigation in PBMC and Caco-2 models, gastrointestinal modifications, and testing the activity of fractions in Caco-2 cells.

### 3.5. Secretion of Cytokines in PBMC After the CJ Fruit Extract Treatment

**Figure 5:**
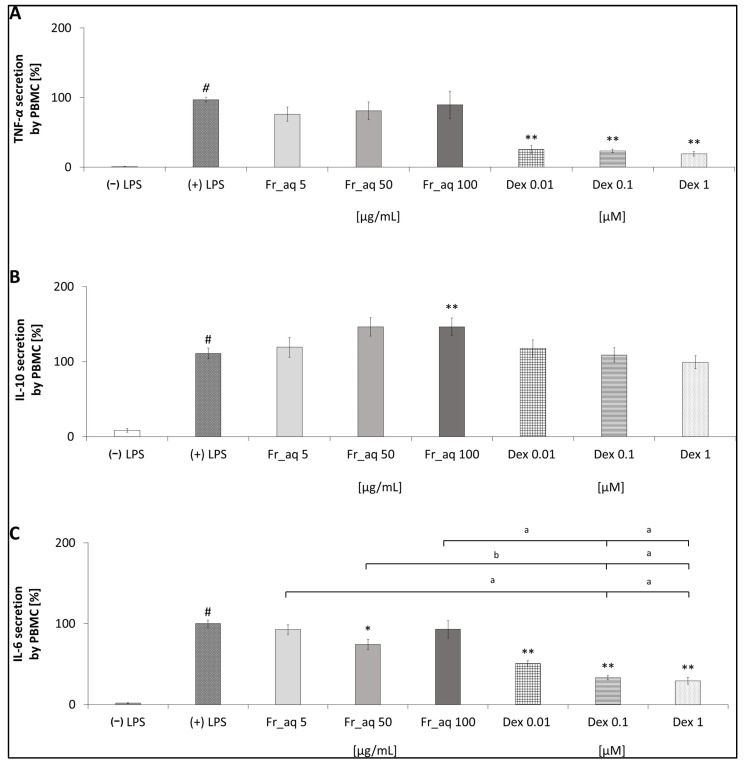
Effect of aqueous (aq) extract from fruits (Fr) of CJ (*Chaenomeles japonica*) on the secretion of TNF-α (**A**), IL-10 (**B**), IL-6 (**C**) in PBMC. Dex—dexamethasone; # *p* < 0.001 vs. (−) LPS; * *p* < 0.05 vs. (+) LPS, ** *p* < 0.001 vs. (+) LPS; ^a^ *p* < 0.001 vs. Dex; ^b^ *p* < 0.05 vs. Dex; a, b—mean the statistical difference between FR_aq and Dex in the indicated concentrations.

In addition to TNF-α (Figure 5A), the release of IL-10 (Figure 5B) and IL-6 (Figure 5C) was screened in the PBMC model after the treatment with CJ aqueous fruit extract. The TNF-α secretion was non-significantly reduced in PBMC, the strongest effect of the extract at 5 μg/mL (76.11 ± 10.20%) and the lowest one at the concentration of 100 μg/mL (89.60 ± 19.61%) was observed. On the other hand, the CJ fruit extract significantly stimulates the secretion of anti-inflammatory cytokines such as IL-10. This activity is even higher than Dex. The CJ fruits at the concentration of 50 μg/mL affected the IL-6 in PBMC in the most significant way; the secretion of this cytokine was 74.27 ± 6.28% (*p* < 0.05). The effect of CJ fruit extract on TNF-α and IL-6 was less relevant than Dex.

### 3.6. Cytokine Secretion in Caco-2 Cells After Treatment with Aqueous Fruit Extract and GI Fractions

#### 3.6.1. IL-8 Secretion

**Figure 6:**
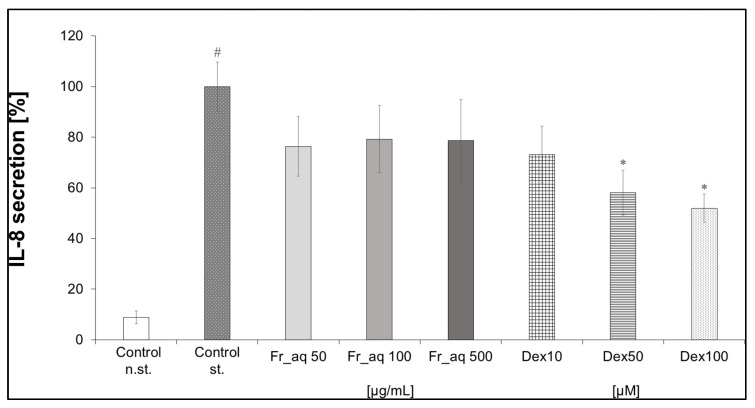
Effect of aqueous (aq) extract from fruits (Fr) of CJ (*Chaenomeles japonica*) on the secretion of IL-8 in Caco-2.; Control st.—[TNF-α (10 ng/mL), IL-1β (25 ng/mL), IFN-γ (10 ng/mL), and LPS (100 ng/mL)]; Dex—dexamethasone; # *p* < 0.001 vs. control n.st.; * *p* < 0.05 vs. control st.

**Figure 7:**
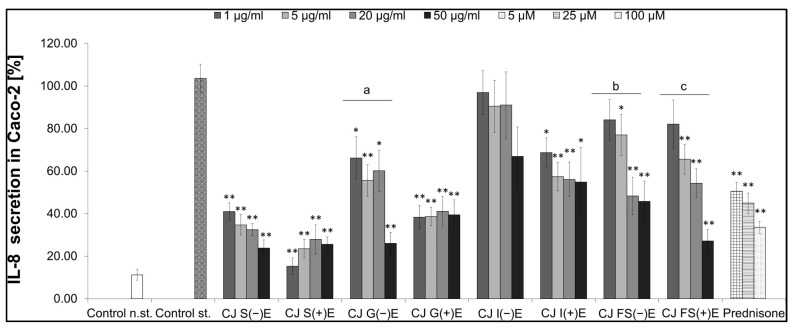
Effect of fractions obtained after gastrointestinal digestion of aqueous CJ fruit extract on the secretion of IL-8 in Caco-2. CJ fractions without digestive enzymes (−)E or with digestive enzymes (+)E: salivary (S), gastric (G), intestinal (I), colon (FS); Control st.—[TNF-α (10 ng/mL), IL-1β (25 ng/mL), IFN-γ (10 ng/mL), and LPS (100 ng/mL)], * *p* < 0.05 vs. (+); ** *p* < 0.001 vs. control st.; a, b, c—the activity of fraction in a concentration-dependent manner.

The secretion of IL-8 in Caco-2 cells was slightly influenced by the aqueous extract of CJ fruits (Figure 6). Considering Caco-2 cells were differentiated into epithelial cells, which are more available for the plant metabolites, the Fr_aq extract was tested in the higher concentration range of 50, 100, and 500 µg/mL than in PMN and PBMC. The activity of the extract was comparable to the effect of Dex in the concentration range from 10 to 100 µM. Next, the CJ aqueous fruit extract was artificially digested in vitro to obtain salivary, gastric, intestinal, and colon fractions in two pathways with or without digestive enzymes (Figure 7).

#### 3.6.2. Activity of GI Fractions from Aqueous CJ Fruit Extract

The most active CJ fractions were S(−)E, S(+)E, G(−)E, and FS(+)E at a concentration of 50 μg/mL; the secretion of IL-8 in Caco-2 cells ranged from 24% to 27% (Figure 7). The overall effect of the S(+)E fraction was the most significant, with the lowest cytokine release at the concentration of 1 μg/mL at 15.35 ± 3.93%, which was more relevant than in the case of prednisone used as a positive control in this experiment. The FS(−)E and FS(+)E fractions affected the IL-8 secretion in Caco-2 cells in a concentration-dependent manner. The IL-8 secretion influenced by the FS(−)E fraction at the highest tested concentration was below 50% compared to the stimulated control. There are relevant differences for the G and I fractions regarding the presence or absence of the enzymes. The cytokine secretion for the G(−)E fraction was 66.10 ± 10.09%, and for the G(+)E was 38.29 ± 5.57% at a concentration of 1 μg/mL, respectively. The I(−)E fraction was a less active (90.47 ± 12.22%) inhibitor for the IL-8 release than the I(+)E fraction (57.37 ± 6.70%) at a 5 μg/mL concentration.

## 4. Discussion

There is an ongoing, increasing need for new, effective, and non-invasive remedies supporting inflammation-related conditions. Natural products in a daily diet as a source of bioactive ingredients are believed to be one of the potential solutions in that field. Bio-assay-guided evaluation of plant products holds the potential for developing anti-inflammatory therapies and optimizing plant processing for nutritional applications. *Chaenomeles japonica*, long used in TCM, has gained recent attention for dietary value, with growing research on its chemical composition and potential use in the food and pharmaceutical industries [2,34]. The full potential of this plant is yet to be determined, as there is a limited amount of publications regarding its activity. There are several studies on the identification of *Chaenomeles japonica* chemical compounds, but the composition differs depending on the part of the plant. There are slight differences in the content of the Japanese quince leaves and fruits, as the procyanidin C1 was found only in leaves, and procyanidins B3 and B1 were identified in fruits [18,35]. On the other hand, CJ seeds are a rich source of fatty acids, hence they are useful for further processing and obtaining new products [2]. The phytochemical composition of leaf and fruit extracts was reported in detail previously [13,31], which was confirmed to a large extent in our study (Figure 2 and Figures S2–S5). Contrary to the report by Turkiewicz et al. (2022) [31], we did not detect any proanthocyanidins in the leaf extracts, while the polyphenols, including flavonoids, are the most abundant compounds (Table 2 and Table 3). Both proanthocyanidins and epicatechin were the most abundant compounds of the aqueous (Table 1) and ethanolic extracts of CJ fruit, which was confirmed by the lack of flavonoid detection and the average content of polyphenols in these extracts (Table 2 and Table 3). As far as CJ seed extracts are concerned, we detected neither chaenomeloside A (28-*O*-β-glucopyranosyl-2α,3β-dihydroxyolean-12-ene-24,28-dioic acid) nor chaenomelogenin A (2α,3β-dihydroxy-12-ene-24,28-dioic acid), which were previously isolated from fruits of *Chaenomeles sinensis* [36]. Contrary to previous reports, ursolic acid, oleanolic acid, and 3-*O*-(E)-3,5-dihydroxycinnamoylursolic acid were not identified in seed extracts [34].

The intercellular cross-talk between intestinal epithelium and immune cells is a crucial factor in the development of inflammation. For this reason, we tested CJ preparations in three models, including PMN, PBMC, and Caco-2 (intestinal epithelium), to evaluate their effects on different cytokine targets maintaining intestinal homeostasis. Their mutual relationship is based on the cytokine network. For example, considering Caco-2 cells, we selected IL-1β to trigger IL-8 release, as the LPS could not have been used for the stimulation. The alkaline phosphatase, which is secreted by the differentiated Caco-2 cells, degrades LPS in differentiated intestinal cells. For this reason, IL-1β was selected as the optimal stimulus because it reliably activated the intestinal epithelial cells, while its secretion could also be quantified in PMN. This approach helped us to explore potential interactions between tissues, suggesting that reduced IL-1β in PMN could limit IL-8 release in the epithelium, which is relevant to inflammatory bowel disease (Table 4) [4].

We previously assessed fruit extract metabolites in the post-digestive fractions [20], which were tested in the direction of potential influence on intestinal inflammation in this research. Our previous report showed that after digestion in vitro, the most abundant constituents of Japanese quince extracts were found to be quite stable during passage through the model gastrointestinal tract. The metabolites such as procyanidin B2 (**6**, [M-H]^−^ *m*/*z* 577), epicatechin (**7**, [M-H]^−^ *m*/*z* 289), and procyanidin trimer (**11**, [M-H]^−^ *m*/*z* 865)were detected (Table 1) in the salivary, gastric, intestinal, and colon fractions. In this in vitro stable model of digestion, the formation of metabolites was observed mainly in the colon fraction incubated with FS. During metabolic transformation, procyanidins tend to polymerize or form different isomers, which arise from the variation in the positioning of monomeric flavan-3-ol units can take place [20].

The epithelial cells, which were gained from Caco-2 cells in vitro in our experiment, are usually exposed to increased levels of phytochemicals. On the other hand, the bioavailability of the compounds found in the plant extract is limited for the immune cells like PBMCs; therefore, the lower concentrations of CJ extract phytochemicals might reach them [37]. The concentrations of plant extracts differ significantly depending on the type of extract and the target cells. Based on the already published papers, the usually tested concentration of plant extracts ranges from 1 µg/mL to 1000 µg/mL. Therefore, this range might be divided into low levels of 1–50 µg/mL, moderate concentrations of 50–200 µg/mL, and high concentrations of 200–1000 µg/mL. We aimed to test a broad range to identify effective and exclude potentially toxic concentrations. It is worth noting that quite high concentrations of plant extracts are used in the antimicrobial assays to establish the minimal inhibitory concentration (MIC). Plant extracts with MICs < 100 μg/mL are classified as highly active antimicrobial agents, while those with MICs ranging from 100 to 500 μg/mL are considered active, and when MICs range between 500 and 1000 μg/mL, they are moderately active [38]. In addition, in the previous reports, the cell lines, e.g., of different colon cell lines (SW-480, HT-29, CCD 841 CoN) were exposed to a different concentration range of CJ phenol-rich leaf extract from 150 to 750 µg/mL [16]. For this reason, the higher concentrations of the extracts used in Caco-2 are justified, considering that intestinal cells in vivo are surrounded by bacteria, and the constituents of the extracts may affect both the immune response of cells and microbiota. This case allows us to indicate the future perspective for considering the activity of the CJ fruit aqueous extract in the microbial environment from the microbiological point of view. The concentrations used in this study (up to 500 µg/mL) were selected to ensure measurable biological effects in in vitro screening assays. We acknowledge that such levels exceed those typically achieved through dietary exposure and should therefore be considered pharmacological rather than nutritional. However, these concentrations may reflect local conditions in the gastrointestinal lumen, where direct contact with plant-derived compounds can be substantially higher than systemic levels.

In our study, whole extracts from different plant parts were considered the potentially active materials. Therefore, we decided to perform the qualitative screening of phytochemicals to select a plant material containing a specific class of compounds. Although the CJ aqueous and ethanolic fruit extracts exerted a comparable effect in general in the preliminary screening in PMN (Figure 4), we decided to use the aqueous fruit extract for further research and processing. Water is the most common solvent, and because of its environmentally friendly aspect, we are trying to follow the new sustainable development trends. It is worth noting that more and more attention is paid to green extraction without using organic solvents in phytochemical procedures [39]. Thus, we determined the aqueous extract to be the primary and universal. Most of the available publications regarding *Chaenomeles japonica* focus on the fruits, as they are the most commonly used part of the plant. The analyses of the CJ fruits demonstrate the presence of compounds with strong biological activity, which was confirmed in our study based on the simultaneous comparison with other plant parts.

In screening the activity of extracts of different parts of Japanese quince (Figure 4), we observed that seed extracts have the most varied effect. The aqueous seed extract tends to stimulate rather than decrease the release of pro-inflammatory cytokines. Taking into consideration the differences between these two seed extracts, we hypothesize that the solvent used for the preparation of the extract can influence the biochemical composition and, in the same way, modify the activity of the seed extracts. Moreover, the stimulating effect of the extract may depend on the presence of fatty acids in the seeds, which are proven to be pro-inflammatory factors [40,41]. Further research concerning the seeds of Japanese quince is needed.

It is said that oxidative stress and inflammation are closely related to pathophysiological processes, which are often simultaneously found in many pathological states. Free radicals can activate NOD-like receptor protein 3 (NLRP3) inflammasome and many transcription factors, among which NF-κB is mainly considered. That leads to the differential expression of some genes involved in inflammatory pathways, production or maturation of cytokines, excessive localized inflammation, and chronic diseases [42]. The radical limitation is one of the factors that might decrease inflammation. It is known that the antioxidant effect depends on polyphenolic compounds such as proanthocyanidins, flavonoids, and phenolic acids. In the case of CJ extracts, such compounds were detected in a few reports. To date, extracts from different parts of CJ have been reported due to their antioxidant properties established in non-cellular models [14,43,44]. To provide a novelty for this study, we focused only on the cytokine secretion. It is worth noting that proanthocyanidins are characterized by a higher number of phenolic moieties in their structures. Considering that the CJ fruit extract exhibited the most promising effect, and proanthocyanidins were mainly detected in the CJ fruit extract, we speculate that the antioxidant effect might primarily justify this effect.

However, proanthocyanidins, having multiple targeting points, influence inflammatory responses on different levels. They have been widely explored in the context of their inhibiting influence on the NF-κB pathway [45]. The NF-κB induces the expression of various pro-inflammatory genes, including those encoding cytokines and chemokines. Proanthocyanidins are known to modulate the NF-κB signaling pathway by preventing IκBα phosphorylation and subsequent NF-κB nuclear translocation, which leads to decreased transcription of pro-inflammatory cytokines. Simultaneously, they attenuate the activation of another pathway—MAPKs, including ERK, JNK, and p38—which further contributes to the downregulation of cytokine expression. Additionally, their antioxidant potential contributes to the suppression of oxidative stress-induced activation of these factors, providing an additional layer of cytokine regulation [8]. Therefore, we hypothesize that Japanese quince-derived compounds detected in the fruit might affect it. It was previously proven that polyphenols found in CJ fruits affect inflammation by downregulating the expression of COX-2 and NF-κB [18]. The CJ leaf extract was recently demonstrated to inhibit the expression of the pro-inflammatory cytokines in LPS-stimulated RAW 264.7 macrophages and downregulate metalloproteinase activity in colon cell lines [16,17]. However, we determined that CJ leaf extract has no relevant influence in inhibiting the secretion of cytokines or chemokines in human neutrophils ex vivo. On the contrary, the CJ ethanolic leaf extract even stimulated the release of IL-8 in a concentration-dependent manner (Figure 4). Although it has been shown that CJ inflorescence tissues extracted with 70% ethanol have an anti-inflammatory activity via inhibition of the 5-lipoxygenase in vitro [43], we did not observe that effect regarding CJ flower extract through cytokines or chemokines secretion. Nevertheless, the discrepancy between studies might be the effect of differences in the model or extraction procedures used. To the best of our knowledge, no data on Japanese quince extracts in ex vivo models are available, and the different parts of the plant have never been compared.

Our last goal was to perform the gastrointestinal digestion in vitro of the aqueous CJ fruit extract rich in proanthocyanidins and obtain fractions for further activity analysis. In the gastrointestinal tract, proanthocyanidin monomers, dimers, and trimers are absorbed into the blood system to a larger extent than oligomeric and polymeric forms due to the decomposition in digestion conditions. The oligomers and polymers can reach the colon, where they are metabolized by gut microbiota and then absorbed as metabolites [37,46]. Thus, digestion can be regarded as the final step of production and food processing. In this study, the digestion-like processing was shown to influence the changes in plant effectiveness. Therefore, detailed knowledge of plant engineering in terms of enhanced nutritional value is important for designing effective plant products [47]. In addition, there is an ongoing need for more eco-friendly solutions in chemistry and the food industry. Enzyme-assisted extraction of bioactive compounds from plant sources is a novel solution, yet not exploited enough. Using this method, higher yields can be produced, with better quality, and it is safe for the environment [39]. Applying enzymes is a new direction for bioengineering modification in plant processing [48]. Therefore, identifying chemical and functional alterations in the CJ fruit compounds after digestion can be crucial to optimizing industrial processing. Multi-stage compartment reactors seem to be a way to find bioactive metabolites, which can be the hydrolysates obtained from alternative sources, such as bacterial strains. Such representatives might be strains of lactic acid bacteria characterized by high proteolytic activity. Enzymatic hydrolysis is a promising method for the production of bioactive compounds. For example, proteolytic hydrolysis with commercial proteases such as pepsin and trypsin is performed to produce bioactive peptides. This approach is considered to develop new functional plants, and metabolites can be target compounds in preventing several diseases [49]. The SHIME (Simulator of the Human Intestinal Microbial Ecosystem) model replicates the entire gastrointestinal tract with control over stirring, pH, and enzymatic changes. The optimal inoculum for accurately mimicking the human gut microbiome remains uncertain. Given the microbial metabolic variation towards polyphenols, SHIME is typically inoculated with a single individual’s faecal microbiota to sustain specific polyphenol transformations [50]. In our study, the inoculum of faecal microbiome, derived from three individuals, was administered to our system (Figure 1). This approach enhanced the strength of reproducing results and supported the generality of IL-8 analysis. The relevant inhibiting activity of the salivary and gastric fractions may depend on the presence of monomers and dimers of epicatechin, catechin, and procyanidin B1. The monomers and dimers of catechin, epicatechin, and procyanidin B1 were demonstrated to permeate across the Caco-2 cells monolayer [51], and each of those substances is an anti-inflammatory agent [52,53,54]. In addition, proanthocyanidins might be metabolized to low molecular weight compounds, such as polyhydroxybenzoic acids, phenolic acids, phenol valerate, and phenol valerolactones [55]. On the other hand, there are noticeable differences in the activity of gastric fractions; the fraction with enzymes inhibited the release of the cytokine more effectively (Figure 7). Although no significant distinction between the chemical composition of both fractions was found in our previous study [20], pepsin could modify the final activity as reported by other authors [56].

Our screening of CJ parts, the first of its kind, identified the fruit, among all plant materials, from a Japanese quince’s shrub, as the most bioactive. Therefore, we confirmed that the fruit is the most valuable for future perspectives of potential applications in the food and pharmaceutical industries. On the other hand, not all examined parts exhibited health-beneficial properties. We emphasize that leaves and seeds display different activity, which requires further investigation. We found that gastrointestinal digestion of CJ extract may modulate its bioactive composition, essential for dietary purposes. However, phenolic inhibition of digestive enzymes may limit enzymatic digestion in our model, and bacterial enzyme activity in the upper gastrointestinal tract—important for phytochemical transformation—was not accounted for. Due to limited amounts of extracts and fractions, the isolation procedures of post-digestive metabolites, which might be of great bioaccessibility and biological significance, were impossible in this research. Thus, further research should focus on quantifying Japanese quince phytochemicals and the engineering of its preparations involving the coculture of plant products with microbiota and modeling an anaerobic environment for potential changes of plant-derived metabolites and their effectiveness. Additionally, using separate in vitro cell models limits the study of intercellular cross-talk, highlighting the need for a more integrated intestinal microenvironment, like a 3D model, to test anti-inflammatory effects. Considering the increasing number of IBD incidences, developing such models is worth investigating for plant products and their post-microbiota-derived metabolites. At present, our research by comparing different CJ plant materials has allowed us to confirm the significance of CJ fruit and its traditional use in Chinese medicine and the dietary industry due to its influence on the epithelial cells and immune response.

## 5. Conclusions

Our study highlights the role of *C. japonica* fruits as the most active plant material from this species, demonstrating their anti-inflammatory effects on cytokine secretion in PMN, PBMC, and Caco-2 cells. On the other hand, it should be emphasized that leaves and seeds display distinct activity patterns compared to fruits, highlighting the need for further studies. In vitro, gastrointestinal digestion revealed that salivary and gastric fractions of the fruit extract are more effective in inhibiting IL-8 secretion than intestinal and colon fractions. The reduced activity of intestinal and colon fractions could stem from complex chemical processes during digestion, with the altered activity of the colon fraction potentially linked to interactions with intestinal microbiota. Although differences in cytokine and chemokine secretion were observed between the enzymatic and non-enzymatic digestion pathways, the findings remain inconclusive and warrant further investigation. On the other hand, the antimicrobial effect of *C. japonica* phytochemicals should be investigated. In addition, conducting in-depth analyses of isolated microbiota-derived metabolites of *C. japonica* to elucidate their roles in modulating inflammation remains challenging.

## Figures and Tables

**Table 1:** Phytochemical profile of CJ aqueous fruit extract.

No.	Compound	Retention Time [min]	λmax [nm]	[M-H]^−^ *m*/*z*	MS/MS
1	*p*-Coumaric acid *O*-hexoside	20.0	319	325	163
2	Apigenin pentoside	20.6	270, 320, 372	447 *	401, 269
3	*p*-Coumaric acid *O*-hexoside	21.4	265, 320	325	163
4	Caffeoyl-di-*O*-galloyl-hexoside	21.8	270, 320	645	627, 528, 493, 451, 289, 193
5	Galloyl derivative of catechin	23.1	270, 320	673	719, 627, 557, 521, 383, 289, 221
6	Procyanidin B2	26.2	276	577	425
7	Epicatechin	28.1	275	289	377, 245, 205
8	Unidentified	29.1	276	567	451, 331
9	Caffeoyl acid derivative	29.7	275	565	403, 223, 179
10	Unidentified	31.3	276	385	341, 217
11	Procyanidin trimer	33.6	254, 354	865	663, 521, 401, 289
12	Feruloylquinic acid	35.6	276	367	191
13	Procyanidin derivative	40.5	280	720	575, 489, 451, 207
14	Unidentified	41.8	276	415	369, 225, 179
15	Procyanidin dimer	43.2	276	577	425
16	Unidentified hexoside	45.9	277, 354	577	431
17	Unidentified	55.8	276	441	395, 305, 225, 179, 161

* +HCOO^−^.

**Table 2:** Total phenol content (TPC) expressed as gallic acid equivalent [mg/g dried plant material]. Lf_et—ethanolic extract of CJ leaves, Lf_aq—aqueous extract of CJ leaves, Fl_et—ethanolic extract of CJ flowers, Fl_aq—aqueous extract of CJ flowers, Sd_et—ethanolic extract of CJ seeds, Sd_aq—aqueous extract of CJ seeds, Fr_et—ethanolic extract of CJ fruits, Fr_aq—aqueous extract of CJ fruits.

Plant Material	Lf_et	Lf_aq	Fl_et	Fl_aq	Sd_et	Sd_aq	Fr_et	Fr_aq
TPC [mg/g]	43.72± 2.61	60.26 ± 3.27	39.97 ± 7.24	39.00 ± 2.41	2.94 ± 0.72	9.58 ± 4.96	24.90 ± 0.72	19.11 ± 1.11

**Table 3:** Total flavonoid content (TFC) expressed as quercetin equivalent [mg/g dried plant material]. Lf_et—ethanolic extract of CJ leaves, Lf_aq—aqueous extract of CJ leaves, Fl_et—ethanolic extract of CJ flowers, Fl_aq—aqueous extract of CJ flowers, Sd_et—ethanolic extract of CJ seeds, Sd_aq—aqueous extract of CJ seeds, Fr_et—ethanolic extract of CJ fruits, Fr_aq—aqueous extract of CJ fruits.

Plant Material	Lf_et	Lf_aq	Fl_et	Fl_aq	Sd_et	Sd_aq	Fr_et	Fr_aq
TFC [mg/g]	26.53± 2.88	15.15 ± 1.09	7.46 ± 2.05	4.78 ± 0.71	n.d.	7.17 ± 2.05	n.d.	n.d.

**Table 4:** The heatmap presents secretion levels relative to a baseline value of 100%. A single ‘+’ indicates approximately 10% inhibition, ‘++’ represents around 20% inhibition, and ‘+++’ corresponds to 30% or greater inhibition. The ‘*’ symbol denotes stimulation of secretion above the baseline level.

Cell Line	TNF-α	IL-1β	IL-8	IL-6	IL-10
PMN	+	+++	++		
PBMC	+			++	*
Caco-2			++		

## Data Availability

Data is contained within the article or Supplementary Materials.

## Supplementary Materials

The following supporting information can be downloaded at: https://www.mdpi.com/article/10.3390/nu17233716/s1. Figure S1 shows the photo of *Chaneomles japonica* var. *alpina* as a source of plant materials. UV chromatograms of the leaf (Figure S2) and flower (Figure S3) extracts of *Chaenomeles japonica* are attached as Supplementary Materials. Figure S4 shows the MS spectra of seed extracts. The description of the figures is included in Tables S1–S3. The UV chromatogram comparing the fruit ethanolic and aqueous extract of *Chaenomeles japonica* is provided in Figure S5.

## Abbreviations

The following abbreviations are used in this manuscript:

CJ	*Chaenomeles japonica*
Dex	Dexamethasone
IBDs	Inflammatory bowel disease
IL-1β	Interleukin 1β
IL-8	Interleukin 8
LPS	Lipopolysaccharide
MIC	Minimal inhibitory concentration
NF-κβ	Nuclear factor κβ
PAMPs	Pathogen-associated molecular patterns
PBMC	Peripheral blood mononuclear cells
PMN	Polymorphonuclear leukocytes
SHIME	Simulator of the Human Intestinal Microbial Ecosystem
TCM	Traditional Chinese Medicine
TNF-α	Tumor necrosis factor α
