# Supplementary material for: Bio-Assay-Guided Study of Chaenomeles japonica–Cytokine Modulation by Fruit Aqueous Extract In Vitro in Connection with Its Processing with Enzymatic and Microbial Additives
Source: Nutrients. 2025 Nov 27;17(23):3716. doi: 10.3390/nu17233716 (PMC12693883; doi:10.3390/nu17233716)
Supplement: Supplementary file 1 [file nutrients-17-03716-s001.zip › nutrients-3983325-supplementary.pdf]

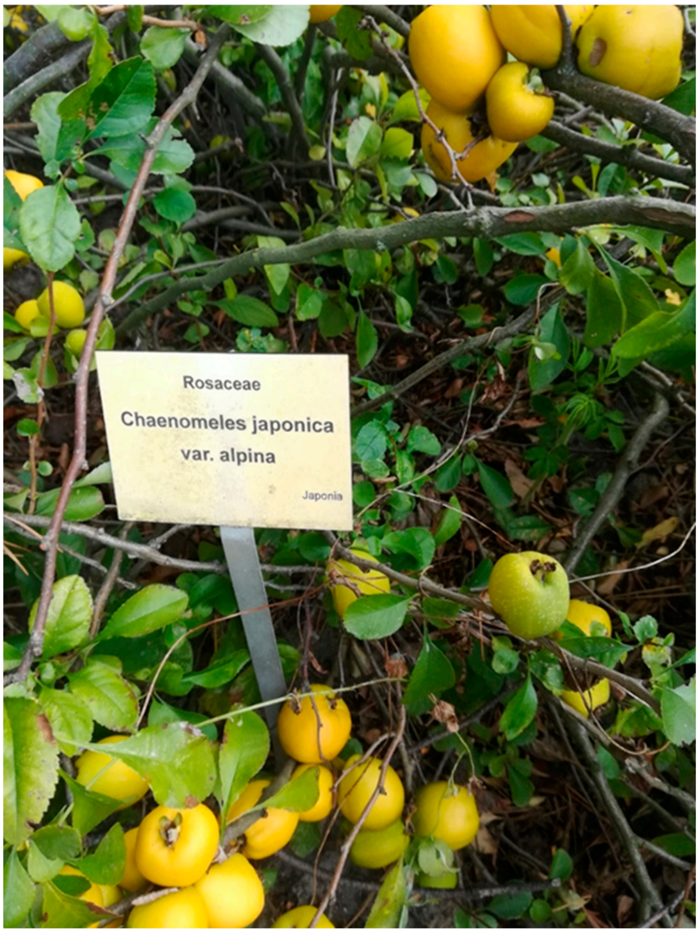

Figure S1. The photo of the shrub of *Chaenomeles japonica* var. *alpina* used in the present study (in the Botanical Garden – of the Center for Biological Diversity Conservation in Powsin, Polish Academy of Sciences, Poland)

HPLC analysis of aqueous-ethanolic and aqueous extracts of plant materials from Chaenomeles japonica

HPLC-DAD-MS^n analysis was performed on a UHPLC-3000 RS system (Dionex, Sunnyvale, California, CA, US) with DAD detection and an AmaZon SL ion trap mass spectrometer with ESI interface (Bruker Daltonik GmbH). Separation was performed on a Zorbax SB-C18 column (150 × 2.1 mm, 1.9 µm, Agilent), set at 25°C. For preliminary phytochemical analysis of extracts, the mobile phase (A) was 0.1% HCOOH in water, and the mobile phase (B) was 0.1% HCOOH in acetonitrile. A linear gradient system was used: 0 – 60 min. 5 – 20% B; 60 – 90 min. 20 – 95% B. The flow rate was 0.2 mL/min. The column was equilibrated for 10 min between injections. UV spectra were recorded over a range of 200 to 450 nm, and chromatograms were acquired at 240, 280, 325, and 350 nm. The LC eluate was introduced directly into the ESI interface without splitting. The nebulizer pressure was 40 psi; the dry gas flow was 9 L/min; the dry temperature was 300°C; and the capillary voltage was 4.5 kV. Analysis was carried out using scans from *m*/*z* 200 to 2,200. Compounds were analysed in negative ion mode. The MS/MS was switched on and tuned to detect neutral loss of 162, 132, 152, 146, and 176 corresponding to the cleavage of sugars or phenolic acid moieties. The MS^2 fragmentation was obtained for the most abundant ion at the time.

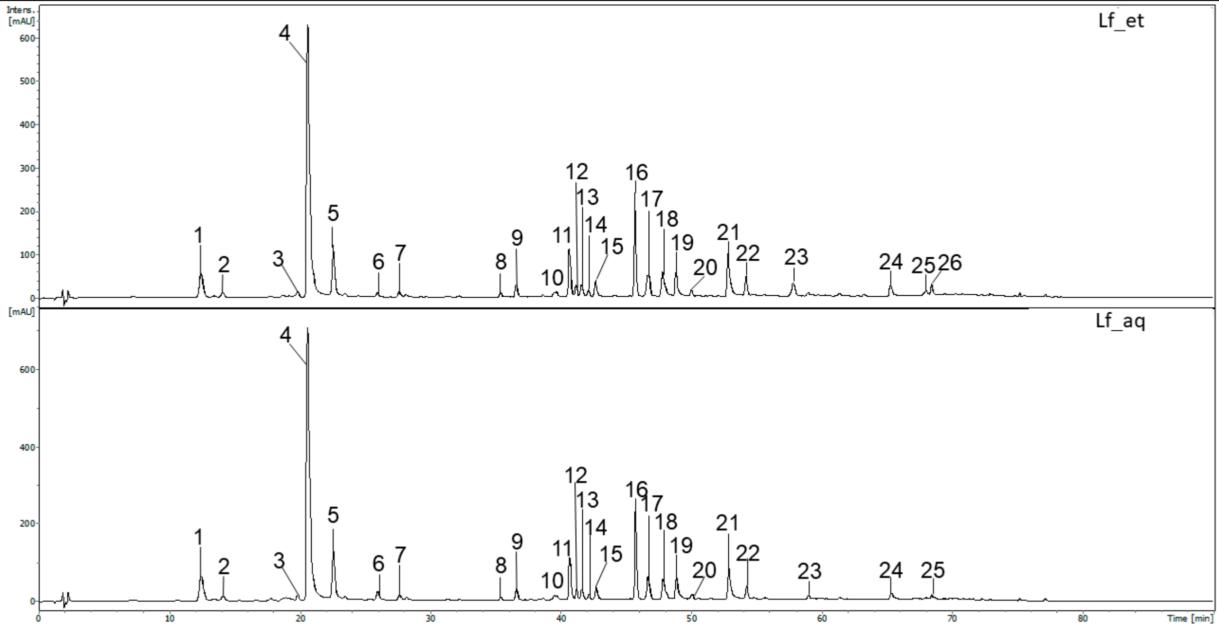

Figure S2. UV chromatogram of ethanolic (Lf_et) and aqueous (Lf_aq) extracts from leaves of CJ (10 mg/mL) registered at λ = 325 nm. The description of numbers assigned to peaks was listed in Table S1.

Table S1. Phytochemical profile of ethanolic (Lf_et) and aqueous (Lf_aq) extracts from leaves of *Chaenomeles japonica*.

No.	Compound	Retention time [min]	λ_{max} [nm]	$[M-H]^-$ *m/z*	MS/MS
1	Caffeoylquinic acid	13.7	323	353	191
2	Pentosyl hexoside of protocatechuic acid	14.8	274	447	404, 315, 153
3	Hexoside of *p*-coumaric acid	20.4	315	325	265, 163
4	Dimer of caffeoylquinic acid	21.3	324	707	353, 191
5	Caffeoylquinic acid	23.2	325	353	191
6	Unidentified	26.2	224, 282	295	173
7	Vomifoliol pentosylhexoside	28.0	281, 311	563*	517, 473, 385, 293, 205
8	Quercetin-*O*-rhamnohexoside	35.7	343	609	463, 301
	overlapped 5-*O*-feruloylquinic acid	35.7		367	191
9	Quercetin-*O*-rhamnohexoside	36.8	352	609	463, 447, 301
	overlapped quercetin rutinoside-rhamnoside	36.8		755	609,
10	Aromadendrin hexoside	40.0	282, 335sh	449	287
11	Kaempferol rutinoside-rhamnoside	41.0	265, 343	739	593, 447, 285
12	Quercetin dirhamnoside	41.4	343	593	447, 301
13	Quercetin *O*-rhamnohexoside	41.8	352	609	463, 301
14	Quercetin hexoside	42.3	266, 343	463	301
15	Quercetin hexoside	43.0	343	463	301
16	Kaempferol dirhamnoside	45.9	264, 341	577	431, 285
17	Naringenin hexoside	46.9	283	433	271
18	Di-*O*-caffeoylquinic acid	47.9	264, 329	515	353, 173
19	Di-*O*-caffeoylquinic acid	49.1	326	515	353, 191
20	Unidentified	50.2	282, 311	563	518, 401, 281, 179
21	Hexoside of caffeoylquinic acid	53.1	326	515	353, 299, 203, 173
22	3-*O*-(deoxy)kaempferol hexoside	54.3	263, 339	431	285
23	Unidentified	58.0	287, 323	697	551, 431, 285
24	Unidentified	65.5	328	457	335, 295, 173
25	Unidentified	68.2	280	447	403, 323, 296
26	Caffeoylquinic acid derivative	68.6	282, 332	677	515, 353

*+HCOOH

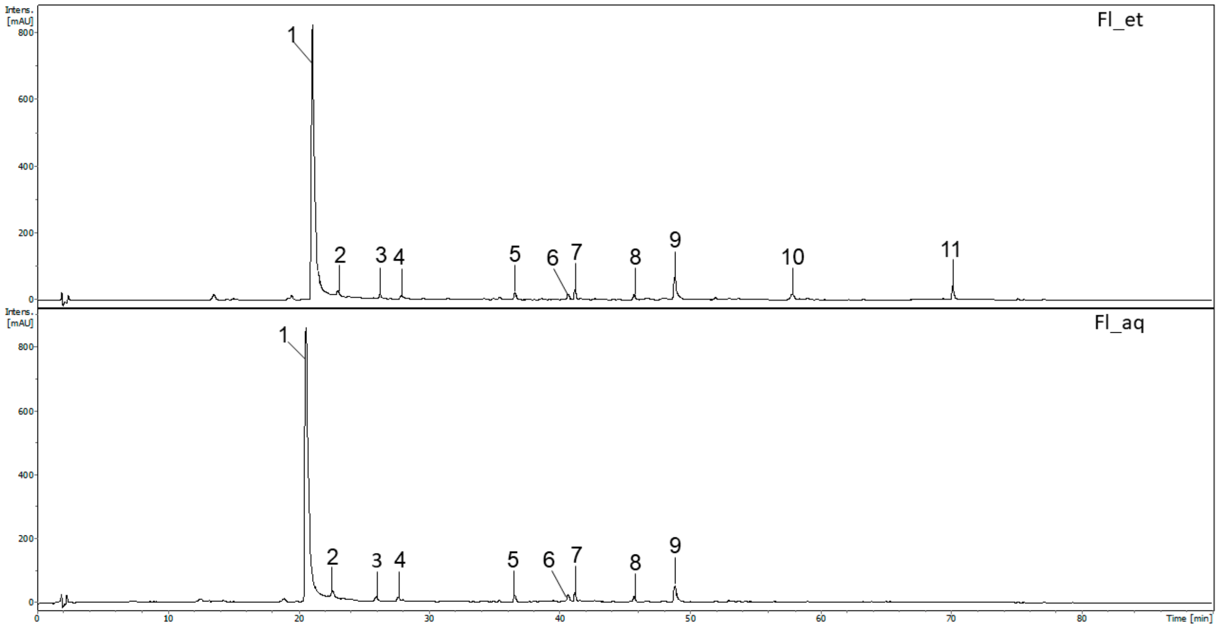

Figure S3. UV chromatogram of ethanolic (Fl_et) and aqueous (Fl_aq) extracts from flowers of CJ (10 mg/mL) registered at λ = 325 nm. The description of numbers assigned to peaks was listed in table S2.

Table S2. Phytochemical profile of ethanolic (Fl_et) and aqueous (Fl_aq) extracts from flowers of *Chaenomeles japonica*.

No.	Compound	Retention time [min]	λ_{max} [nm]	$[M-H]^-$ *m/z*	MS/MS
1	Dimer of caffeoylquinic acid	21.3	239, 324	707	353, 191
2	Gallic acid derivative	23.1	325	340	294, 188, 161
3	Caffeoylquinic acid	26.5	281, 320	353	191, 179
4	Epicatechin	28.1	281	289	245, 205, 109
5	Quercetin-*O*-rhamnohexoside	36.7	352	609	755, 463, 301
6	Kaempferol 3-*O*-(*p*-coumaroyl)hexoside	40.8	265, 350	593	739, 447, 285
7	Quercetin dirhamnoside	41.4	352	593	447, 301
8	Kaempferol dirhamnoside	45.9	347	577	431, 285
9	Di-*O*-caffeoylquinic acid	49.0	327	515	353, 191, 179
10	Epi(catechin) rhamnohexoside	57.6	329	667	497, 377, 289
11	Unidentified	70.3	287	582	462, 342, 253

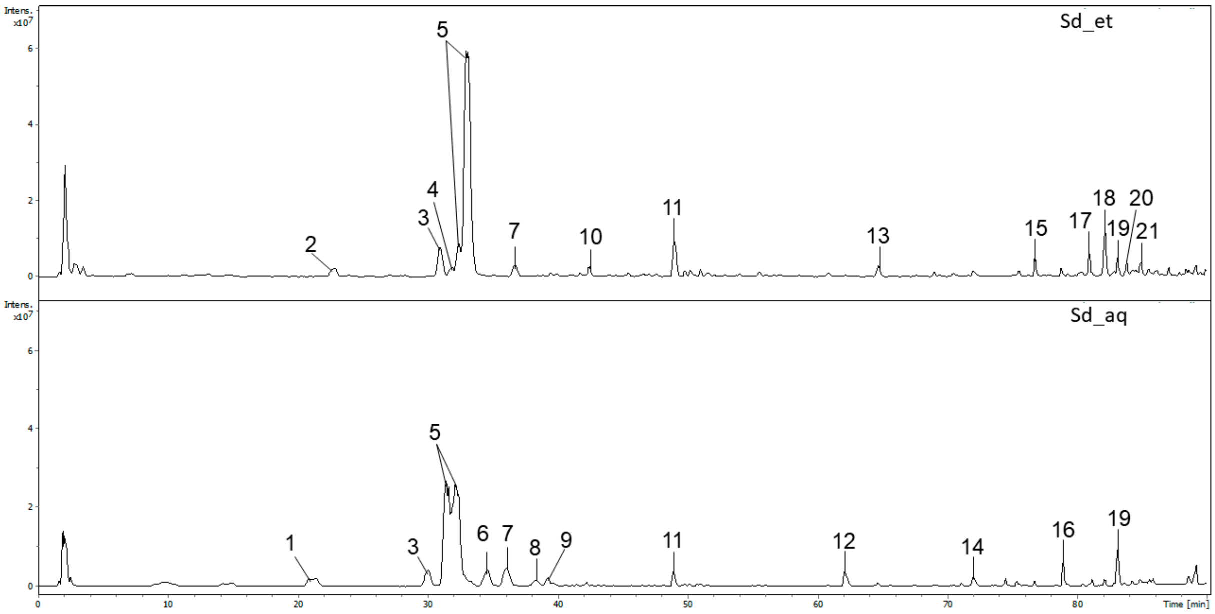

Figure S4. MS spectrum of ethanolic (Sd_et) and aqueous (Sd_aq) extracts from seeds of CJ (10 mg/mL). The description of numbers assigned to peaks was listed in table S3.

Table S3. Spectral data of ethanolic (Sd_et) and aqueous (Sd_aq) extracts from seeds of *Chaenomeles japonica*.

No.	Compound	Retention time [min]	$[M-H]^-$ *m/z*	MS/MS
1	Acetyl derivative of genistin	20.9	475	431, 269, 161
2	Acetyl derivative of genistin	22.8	475	431, 269, 161
3	Genistin	30.0	477*	431, 269
4	Hexoside of amygdalin	31.8	618	456
5	Amygdalin	32.2	456	323, 179, 161
6	Unidentified	34.6	664*	618, 485, 383, 323, 221
7	Unidentified	36.5	664*	618, 485, 383, 323, 263
8	Hexoside of amygdalin	38.3	618	456
9	Amygdalin derivative	39.3	664	618, 502, 456
10	Unidentified	42.5	593*	547, 385, 223
11	Unidentified	49.0	475*	429, 325, 265, 163
12	Unidentified	62.1	369*	323, 295, 233
13	Unidentified	64.7	557*	511, 179
14	Unidentified	72.0	329	311, 228, 211, 171
15	Terpene derivative	76.7	517	455
16	Unidentified	78.9	313	295, 183
17	Unidentified	81.0	723*	677, 415, 397
18	Unidentified	82.1	485	467, 423, 405, 337
19	Unidentified	83.1	295	277, 171
20	Unidentified	83.8	561*	515, 279, 235
21	Unidentified	84.9	297	279, 251, 155

*+HCOOH

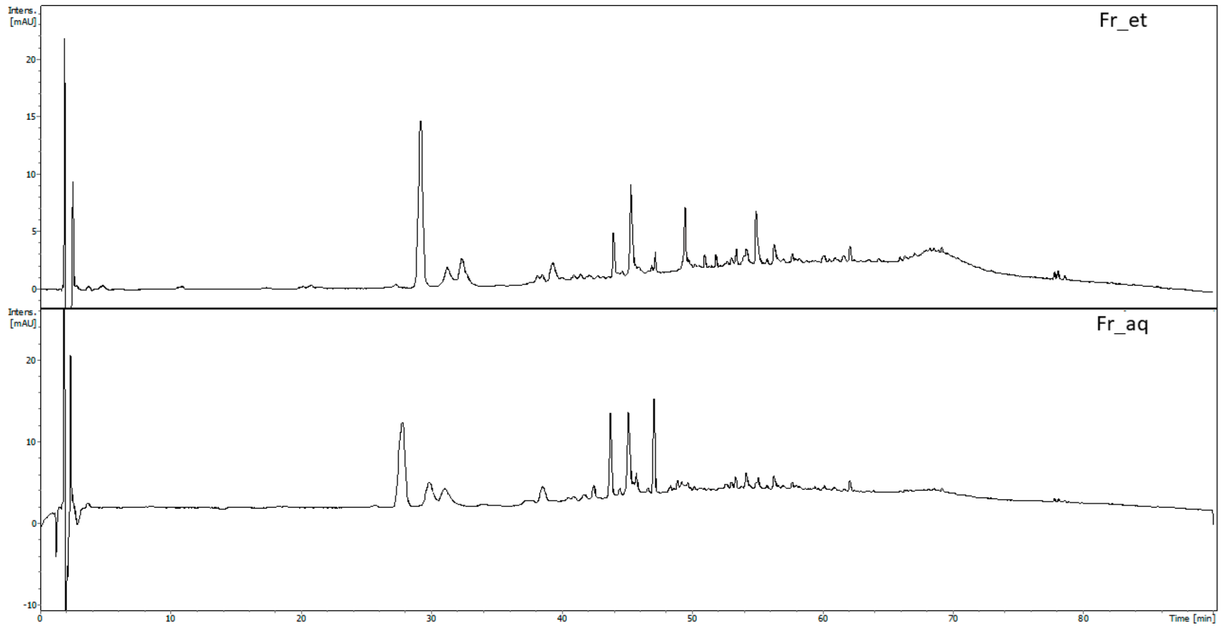

Figure S5. UV chromatogram of ethanolic (Fr_et) and aqueous (Fr_aq) extracts from fruits of CJ (10 mg/mL) registered at λ = 325 nm. The phytochemical analysis of aqueous extract was provided by Siegień et al. 2021 and in the main text.
